# Tetra­kis(2-amino-5-chloro­pyridinium) di­hydrogen cyclo­hexa­phosphate

**DOI:** 10.1107/S1600536814003584

**Published:** 2014-02-22

**Authors:** Ahmed Hamdi, Lamia Khederi, Mohamed Rzaigui

**Affiliations:** a Chemistry Laboratory of Materials, Sciences Faculty of Bizerta, 7021 Jarzouna, Bizerta, Tunisia

## Abstract

In the crystal structure of the title compound, 4C_5_H_6_ClN_2_^+^·H_2_P_6_O_18_^4−^, the [H_2_P_6_O_18_]^4−^ anions are interconnected by O—H⋯O hydrogen bonds, leading to the formation of infinite ribbons extending along the *a-*axis direction. These ribbons are linked to the organic cations, *via* N—H⋯O and C—H⋯O hydrogen bonds, into a three-dimensional network. The six P atoms of the [H_2_P_6_O_18_]^4−^ anion form a chair conformation. The complete cyclohexaphosphate anion is generated by inversion symmetry.

## Related literature   

For properties of hybrid materials, see: Ozin (1992 ▶); Teraski *et al.* (1987 ▶). For related structures containing cyclo­hexa­phosphate rings, see: Bel Haj Salah *et al.* (2014 ▶); Khedhiri *et al.* (2007 ▶, 2012 ▶); Amri *et al.* (2009 ▶); Abid *et al.* (2012 ▶). For bond lengths in pyridine, see: Bak *et al.* (1959 ▶); Hemissi *et al.* (2010 ▶); Toumi Akriche *et al.* (2010 ▶); Akriche & Rzaigui (2005 ▶); Janiak (2000 ▶). For the preparation of cyclo­hexa­phospho­ric acid, see: Schulke & Kayser (1985 ▶).
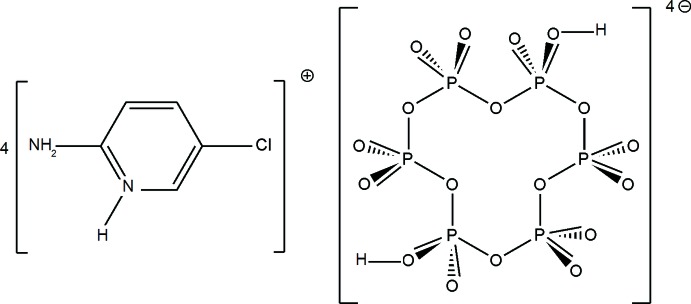


## Experimental   

### 

#### Crystal data  

4C_5_H_6_ClN_2_^+^·H_2_O_18_P_6_^4−^
*M* *_r_* = 994.11
Triclinic, 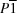

*a* = 9.199 (3) Å
*b* = 9.304 (2) Å
*c* = 11.327 (3) Å
α = 74.98 (3)°
β = 85.17 (2)°
γ = 75.20 (2)°
*V* = 905.1 (5) Å^3^
*Z* = 1
Ag *K*α radiation
λ = 0.56087 Å
μ = 0.35 mm^−1^
*T* = 293 K
0.32 × 0.22 × 0.15 mm

#### Data collection  

Enraf–Nonius CAD-4 diffractometer
11291 measured reflections
8865 independent reflections
5387 reflections with *I* > 2σ(*I*)
*R*_int_ = 0.020
2 standard reflections every 120 min intensity decay: 1%

#### Refinement  

*R*[*F* ^2^ > 2σ(*F* ^2^)] = 0.056
*wR*(*F* ^2^) = 0.155
*S* = 1.02
8865 reflections
305 parameters
H atoms treated by a mixture of independent and constrained refinement
Δρ_max_ = 0.82 e Å^−3^
Δρ_min_ = −0.65 e Å^−3^

### 

Data collection: *CAD-4 EXPRESS* (Enraf–Nonius, 1994 ▶); cell refinement: *CAD-4 EXPRESS*; data reduction: *XCAD4* (Harms & Wocadlo, 1995 ▶); program(s) used to solve structure: *SHELXS97* (Sheldrick, 2008 ▶); program(s) used to refine structure: *SHELXL97* (Sheldrick, 2008 ▶); molecular graphics: *ORTEP-3 for Windows* (Farrugia, 2012 ▶); software used to prepare material for publication: *WinGX* (Farrugia, 2012 ▶).

## Supplementary Material

Crystal structure: contains datablock(s) I. DOI: 10.1107/S1600536814003584/fj2662sup1.cif

Structure factors: contains datablock(s) I. DOI: 10.1107/S1600536814003584/fj2662Isup2.hkl

CCDC reference: 987420

Additional supporting information:  crystallographic information; 3D view; checkCIF report

## Figures and Tables

**Table 1:** Hydrogen-bond geometry (Å, °)

*D*—H⋯*A*	*D*—H	H⋯*A*	*D*⋯*A*	*D*—H⋯*A*
N1—H1⋯O5^i^	0.96 (3)	1.78 (3)	2.736 (3)	177 (3)
O1—H1*A*⋯O8^ii^	0.78 (5)	1.66 (5)	2.418 (3)	165 (5)
N2—H2*A*⋯O6^i^	0.82 (4)	2.02 (4)	2.844 (3)	173 (3)
N2—H2*B*⋯O9^iii^	0.80 (3)	2.29 (3)	3.000 (3)	149 (4)
N3—H3⋯O2	0.78 (4)	2.03 (3)	2.781 (3)	161 (3)
N4—H4*A*⋯O2	0.80 (3)	2.57 (3)	3.179 (3)	134 (2)
N4—H4*A*⋯O6	0.80 (3)	2.16 (3)	2.827 (3)	142 (3)
N4—H4*B*⋯O9^iv^	0.93 (4)	1.95 (4)	2.852 (3)	162 (3)
C5—H5⋯O5^v^	0.87 (3)	2.51 (3)	3.322 (3)	157 (3)
C10—H10⋯O1^ii^	0.96 (4)	2.42 (4)	3.262 (3)	146 (3)

Symmetry codes: (i) 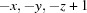; (ii) 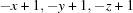; (iii) 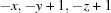; (iv) 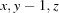; (v) 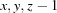.

**Fig. 1.**
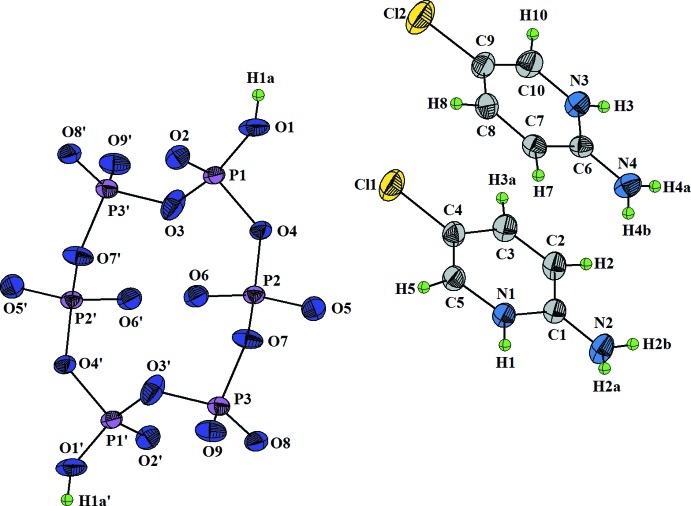
ORTEP drawing of the cyclic anion [H2P6O18]4- and the two independent 2-amino-5-chloropyridinium cations. Displacement ellipsoids for non H-atoms are drawn at the 40% probability level. [Symmetry code: (i) x, y, z]

**Fig. 2.**
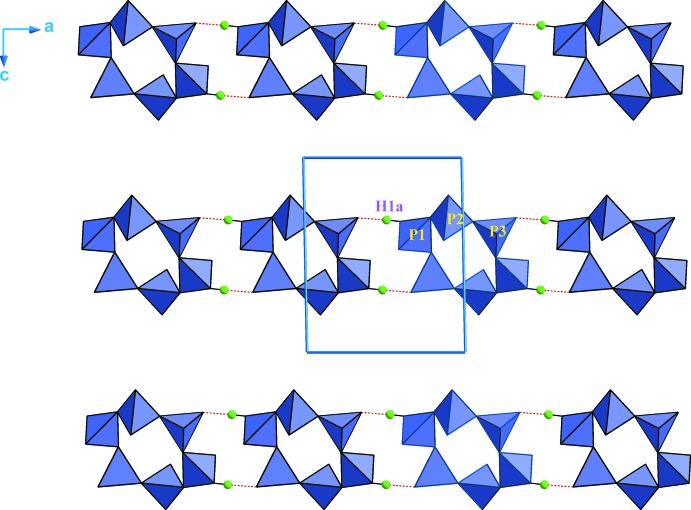
Projection of the [H2P6O18]4- anions running along the a axis

**Fig. 3.**
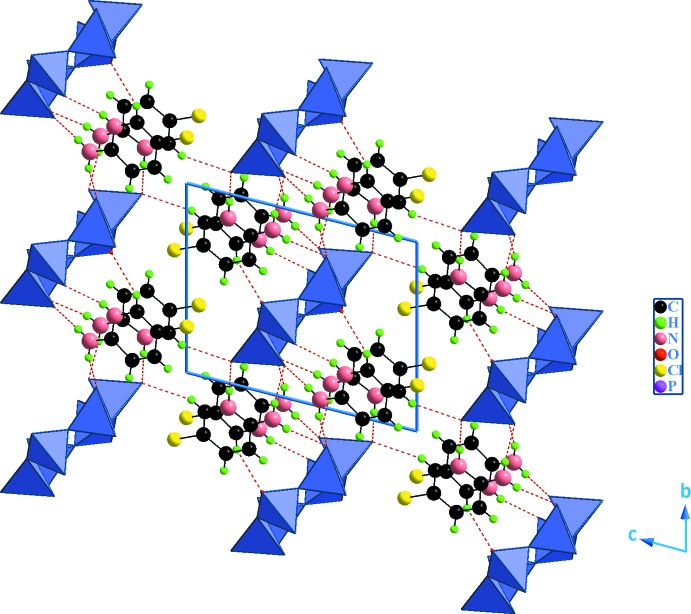
Projection of the structure along the a direction

**Fig. 4.**
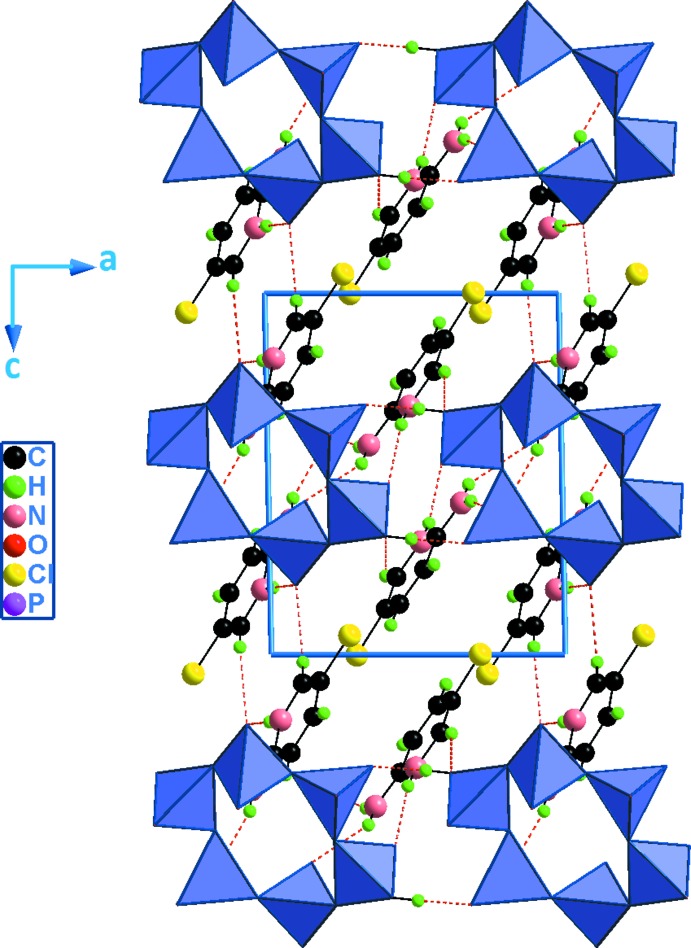
Projection of the structure along the b direction

## supplementary crystallographic information

### 1. Comment

Research in organic-inorganic materials has experienced considerable growth in recent years for the purpose of generating desirable properties and functionalities. An important strategy employed in studying such systems has been to take advantage of hydrogen-bond interactions between organic cations and inorganic anions, since they have been recognized as the most powerful force to generate supramolecular network in one, two and three dimensions (Ozin, 1992, Teraski *et al.*, 1987).

In this context, our aims have been focused on the organic salts of cyclohexaphosphates systems. The title compound (I) provides another example of these kinds of materials.

The partial three-dimensional plot in Figure 1 illustrates the geometrical configuration of the [H_2_P_6_O_18_]^4-^ ring and the two independent organic cations [C_5_H_6_ClN_2_]^+^ in the (I) structure. The dihydrogen-cyclohexaphosphate anions are connected through strong hydrogen bonds characterized by short distances (d_O···O_ = 2.418 (3) Å) leading to the formation of infinite and parallel [H_2_P_6_O_18_]_n_^4-^ slabs (Figure 2). It is worth noting that the strong H-bond between phosphoric rings (Table 1) (d_O···O_ < 2.73 Å) is rather observed in cyclohexaphosphates.

Two crystallographically independent cations coexist in this structure. They are arranged in pairs and anchored onto the anionic ribbons *via* N—H···O and C—H···O hydrogen bonds to keep up the three-dimensional network cohesion (Figure 3, Figure 4).

The [H_2_P_6_O_18_]^4-^, group with chair conformation shows its standard geometry, the longest bonds length ranging between 1.580 (2) and 1.608 (2) Å, correspond to the bridging oxygen atom, the intermediate one, P1—O1 = 1.502 (2) Å, correspond to the P—OH bonding and the shortest ones spreading between 1.460 (2) and 1.498 (2) Å, correspond to the external oxygen atoms. The P—P—P angles of 111.0 (1), 120.5 (1) and 125.1 (4)° show that the rings are slightly distorted from the ideal threefold symmetry. The P—P distances as well as P—O—P or O—P—O angles show that these features are similar to those commonly observed in condensed phosphate anions (Bel Haj Salah *et al.*, 2014, Khedhiri *et al.*, 2012, Khedhiri *et al.*, 2007, Amri *et al.*, 2009, Abid *et al.*, 2012).

Despite the limited number of organic cation cyclohexaphosphates (about forty related structures), we can distinguish only few acidic cyclohexaphosphates such as the title compound (I).

The examination of pyridinium rings shows that these units are planar with mean deviation of 0.0036 and 0.0038 Å from least-square plane defined by the six constituent atoms. The average C—N distances in pyridinium rings is 1.353 Å and the C—C bond lengths are 1.380 Å. The latter value, being shorter than 1.39–1.41 Å, reported for non-substituent pyridine, may indicate some aromatic bond characters (Bak *et al.*, 1959). These values are in accordance with those observed in others compounds (Hemissi *et al.*, 2010, Toumi Akriche *et al.*, 2010, Akriche *et al.*, 2005). The inter-planar distance between the pyridine rings is in the vicinity of 4.00 Å, which is significantly longer than 3.80 Å for the *p*-p interaction (Janiak, 2000). In addition to electrostatic and van der Waals interactions, the structure is further stabilized with a three-dimensional network of O—H···O, N—H···O and the weaker C—H···O hydrogen bonds (Table 1, Figure 3).

### 2. Experimental

Single crystals of the title compound were prepared in two steps. In the first one, 50 ml of an aqueous solution of cyclohexaphosphoric acid was prepared by protonation of 4 g of Li_6_P_6_O_18_, obtained by the Schulke process (Schulke *et al.*, 1985), with an ion exchange resin (Amberlite IR 120). In the second one, the frech acidic solution (20 ml, 2.6 mmol) was immediately neutralized with a solution of 2-amino-5-chloropyridine (2.8 mmol in 10 ml of ehanol) under continuous stirring. Good quality of prismatic-shaped crystals were obtained after a slow evaporation during few days at ambient temperature

### 3. Refinement

All H atoms were found in difference Fourier synthesis and refined in isotropic approximation

### Crystal data

**Table:**

4C_5_H_6_ClN_2_^+^·H_2_O_18_P_6_^4^^−^	*Z* = 1
*M**_r_* = 994.11	*F*(000) = 504
Triclinic, *P*1	*D*_x_ = 1.824 Mg m^−^^3^
Hall symbol: -P 1	Ag *K*α radiation, λ = 0.56087 Å
*a* = 9.199 (3) Å	Cell parameters from 25 reflections
*b* = 9.304 (2) Å	θ = 9–11°
*c* = 11.327 (3) Å	µ = 0.35 mm^−^^1^
α = 74.98 (3)°	*T* = 293 K
β = 85.17 (2)°	Rectangular, colorless
γ = 75.20 (2)°	0.32 × 0.22 × 0.15 mm
*V* = 905.1 (5) Å^3^	

### Data collection

**Table:**

Enraf–Nonius CAD-4 diffractometer	*R*_int_ = 0.020
Radiation source: fine-focus sealed tube	θ_max_ = 28.0°, θ_min_ = 2.1°
Graphite monochromator	*h* = −15→15
non–profiled ω scans	*k* = −15→15
11291 measured reflections	*l* = −18→3
8865 independent reflections	2 standard reflections every 120 min
5387 reflections with *I* > 2σ(*I*)	intensity decay: 1%

### Refinement

**Table:**

Refinement on *F*^2^	Primary atom site location: structure-invariant direct methods
Least-squares matrix: full	Secondary atom site location: difference Fourier map
*R*[*F*^2^ > 2σ(*F*^2^)] = 0.056	Hydrogen site location: inferred from neighbouring sites
*wR*(*F*^2^) = 0.155	H atoms treated by a mixture of independent and constrained refinement
*S* = 1.02	*w* = 1/[σ^2^(*F*_o_^2^) + (0.0798*P*)^2^ + 0.0876*P*] where *P* = (*F*_o_^2^ + 2*F*_c_^2^)/3
8865 reflections	(Δ/σ)_max_ = 0.001
305 parameters	Δρ_max_ = 0.82 e Å^−^^3^
0 restraints	Δρ_min_ = −0.65 e Å^−^^3^

### Special details

**Table:**

Geometry. All e.s.d.'s (except the e.s.d. in the dihedral angle between two l.s. planes) are estimated using the full covariance matrix. The cell e.s.d.'s are taken into account individually in the estimation of e.s.d.'s in distances, angles and torsion angles; correlations between e.s.d.'s in cell parameters are only used when they are defined by crystal symmetry. An approximate (isotropic) treatment of cell e.s.d.'s is used for estimating e.s.d.'s involving l.s. planes.
Refinement. Refinement of *F*^2^ against ALL reflections. The weighted *R*-factor *wR* and goodness of fit *S* are based on *F*^2^, conventional *R*-factors *R* are based on *F*, with *F* set to zero for negative *F*^2^. The threshold expression of *F*^2^ > σ(*F*^2^) is used only for calculating *R*-factors(gt) *etc*. and is not relevant to the choice of reflections for refinement. *R*-factors based on *F*^2^ are statistically about twice as large as those based on *F*, and *R*- factors based on ALL data will be even larger.

### Fractional atomic coordinates and isotropic or equivalent isotropic displacement parameters (Å^2^)

**Table:**

	*x*	*y*	*z*	*U*_iso_*/*U*_eq_	
P1	0.31788 (5)	0.39966 (6)	0.59770 (5)	0.0246 (1)	
P2	0.09221 (5)	0.22559 (6)	0.68658 (5)	0.0249 (1)	
P3	0.21377 (5)	0.64715 (6)	0.38126 (5)	0.0261 (1)	
O1	0.39658 (19)	0.4679 (2)	0.67218 (19)	0.0454 (6)	
O2	0.40900 (17)	0.29457 (17)	0.52877 (14)	0.0335 (4)	
O3	0.20301 (19)	0.5379 (2)	0.51466 (15)	0.0455 (5)	
O4	0.20430 (16)	0.32887 (17)	0.69374 (13)	0.0297 (4)	
O5	0.08853 (19)	0.11700 (18)	0.80701 (15)	0.0375 (5)	
O6	0.12380 (17)	0.16793 (19)	0.57518 (15)	0.0353 (4)	
O7	0.06189 (16)	0.64266 (17)	0.32794 (16)	0.0359 (5)	
O8	0.33616 (16)	0.56632 (18)	0.30855 (14)	0.0333 (4)	
O9	0.21174 (18)	0.80107 (18)	0.39100 (17)	0.0407 (5)	
Cl1	0.26706 (10)	0.37124 (10)	−0.05308 (6)	0.0606 (3)	
N1	0.0267 (2)	0.1302 (2)	0.18049 (17)	0.0341 (5)	
N2	−0.0748 (3)	0.1321 (3)	0.3732 (2)	0.0430 (7)	
C1	0.0017 (3)	0.1920 (2)	0.2777 (2)	0.0319 (5)	
C2	0.0596 (3)	0.3211 (3)	0.2711 (2)	0.0382 (7)	
C3	0.1390 (3)	0.3761 (3)	0.1712 (2)	0.0396 (7)	
C4	0.1628 (3)	0.3061 (3)	0.0732 (2)	0.0377 (6)	
C5	0.1044 (3)	0.1844 (3)	0.0791 (2)	0.0376 (6)	
Cl2	0.71034 (11)	0.24071 (11)	−0.00108 (7)	0.0685 (3)	
N3	0.4844 (2)	0.2016 (2)	0.31254 (19)	0.0370 (6)	
N4	0.3444 (2)	0.0352 (2)	0.4197 (2)	0.0387 (6)	
C6	0.4320 (2)	0.0752 (2)	0.3261 (2)	0.0298 (5)	
C7	0.4751 (2)	−0.0097 (2)	0.2364 (2)	0.0325 (6)	
C8	0.5622 (3)	0.0394 (3)	0.1400 (2)	0.0373 (6)	
C9	0.6083 (3)	0.1751 (3)	0.1271 (2)	0.0405 (7)	
C10	0.5705 (3)	0.2533 (3)	0.2150 (2)	0.0434 (7)	
H1A	0.483 (5)	0.441 (5)	0.680 (4)	0.113 (17)*	
H1	−0.010 (3)	0.041 (3)	0.186 (3)	0.047 (8)*	
H2	0.041 (3)	0.362 (3)	0.333 (3)	0.053 (9)*	
H2A	−0.089 (3)	0.046 (4)	0.382 (3)	0.044 (8)*	
H2B	−0.083 (4)	0.164 (4)	0.433 (3)	0.055 (9)*	
H3A	0.174 (3)	0.469 (4)	0.162 (3)	0.055 (9)*	
H5	0.107 (3)	0.139 (4)	0.021 (3)	0.053 (9)*	
H3	0.457 (4)	0.247 (4)	0.363 (3)	0.054 (9)*	
H4A	0.319 (3)	0.082 (3)	0.471 (2)	0.033 (7)*	
H4B	0.312 (4)	−0.053 (5)	0.425 (3)	0.075 (11)*	
H7	0.442 (3)	−0.106 (3)	0.249 (3)	0.044 (8)*	
H8	0.592 (3)	−0.006 (3)	0.082 (2)	0.031 (6)*	
H10	0.607 (4)	0.340 (4)	0.218 (3)	0.058 (9)*	

### Atomic displacement parameters (Å^2^)

**Table:**

	*U*^11^	*U*^22^	*U*^33^	*U*^12^	*U*^13^	*U*^23^
P1	0.0202 (2)	0.0277 (2)	0.0285 (2)	−0.0090 (2)	0.0034 (2)	−0.0097 (2)
P2	0.0230 (2)	0.0271 (2)	0.0294 (2)	−0.0115 (2)	0.0022 (2)	−0.0111 (2)
P3	0.0231 (2)	0.0274 (2)	0.0304 (2)	−0.0090 (2)	−0.0001 (2)	−0.0093 (2)
O1	0.0284 (7)	0.0669 (11)	0.0609 (12)	−0.0270 (8)	0.0119 (7)	−0.0393 (10)
O2	0.0306 (7)	0.0335 (7)	0.0360 (8)	−0.0039 (6)	0.0058 (6)	−0.0135 (6)
O3	0.0387 (8)	0.0452 (9)	0.0345 (8)	0.0062 (7)	0.0107 (7)	0.0020 (7)
O4	0.0297 (6)	0.0388 (7)	0.0294 (7)	−0.0210 (6)	0.0047 (5)	−0.0129 (6)
O5	0.0454 (9)	0.0366 (8)	0.0343 (8)	−0.0224 (7)	−0.0015 (7)	−0.0028 (6)
O6	0.0351 (7)	0.0438 (8)	0.0376 (8)	−0.0184 (6)	0.0078 (6)	−0.0226 (7)
O7	0.0243 (6)	0.0320 (7)	0.0589 (10)	−0.0077 (5)	−0.0042 (6)	−0.0224 (7)
O8	0.0254 (6)	0.0452 (8)	0.0339 (8)	−0.0124 (6)	0.0046 (5)	−0.0158 (7)
O9	0.0367 (8)	0.0351 (8)	0.0601 (11)	−0.0175 (6)	−0.0027 (7)	−0.0196 (7)
Cl1	0.0841 (5)	0.0739 (5)	0.0387 (3)	−0.0499 (4)	0.0169 (3)	−0.0155 (3)
N1	0.0451 (10)	0.0335 (8)	0.0322 (9)	−0.0198 (8)	0.0038 (7)	−0.0142 (7)
N2	0.0648 (14)	0.0380 (10)	0.0362 (10)	−0.0264 (10)	0.0150 (9)	−0.0182 (9)
C1	0.0409 (10)	0.0271 (8)	0.0313 (10)	−0.0126 (8)	0.0012 (8)	−0.0100 (7)
C2	0.0574 (14)	0.0313 (10)	0.0330 (11)	−0.0199 (10)	0.0031 (10)	−0.0127 (8)
C3	0.0558 (14)	0.0339 (10)	0.0365 (11)	−0.0217 (10)	0.0022 (10)	−0.0120 (9)
C4	0.0482 (12)	0.0406 (11)	0.0300 (10)	−0.0220 (10)	0.0017 (9)	−0.0086 (9)
C5	0.0477 (12)	0.0426 (11)	0.0323 (10)	−0.0222 (10)	0.0044 (9)	−0.0169 (9)
Cl2	0.0848 (6)	0.0781 (5)	0.0483 (4)	−0.0413 (5)	0.0239 (4)	−0.0125 (4)
N3	0.0453 (10)	0.0351 (9)	0.0389 (10)	−0.0173 (8)	0.0078 (8)	−0.0190 (8)
N4	0.0389 (10)	0.0384 (10)	0.0450 (11)	−0.0143 (8)	0.0103 (8)	−0.0197 (9)
C6	0.0299 (9)	0.0282 (8)	0.0343 (10)	−0.0084 (7)	−0.0007 (7)	−0.0114 (7)
C7	0.0354 (10)	0.0296 (9)	0.0368 (10)	−0.0082 (8)	−0.0022 (8)	−0.0147 (8)
C8	0.0414 (11)	0.0421 (11)	0.0333 (11)	−0.0111 (9)	0.0019 (9)	−0.0177 (9)
C9	0.0447 (12)	0.0461 (12)	0.0333 (11)	−0.0171 (10)	0.0064 (9)	−0.0107 (10)
C10	0.0554 (14)	0.0379 (11)	0.0444 (13)	−0.0242 (10)	0.0065 (11)	−0.0127 (10)

### Geometric parameters (Å, º)

**Table:**

Cl1—C4	1.718 (3)	N3—C6	1.350 (3)
Cl2—C9	1.722 (3)	N3—C10	1.361 (3)
P1—O2	1.4612 (17)	N4—C6	1.310 (3)
P1—O1	1.501 (2)	N3—H3	0.78 (4)
P1—O3	1.5816 (19)	N4—H4B	0.93 (4)
P1—O4	1.5804 (17)	N4—H4A	0.80 (3)
P2—O4	1.5981 (17)	C1—C2	1.416 (4)
P2—O7^i^	1.6082 (17)	C2—C3	1.352 (3)
P2—O5	1.4758 (18)	C3—C4	1.402 (3)
P2—O6	1.4744 (18)	C4—C5	1.357 (4)
P3—O3	1.5989 (18)	C2—H2	0.87 (3)
P3—O9	1.4599 (18)	C3—H3A	0.98 (4)
P3—O7	1.5823 (17)	C5—H5	0.87 (3)
P3—O8	1.4979 (17)	C6—C7	1.415 (3)
O1—H1A	0.78 (5)	C7—C8	1.351 (3)
N1—C5	1.353 (3)	C8—C9	1.401 (4)
N1—C1	1.347 (3)	C9—C10	1.353 (4)
N2—C1	1.317 (3)	C7—H7	0.99 (3)
N1—H1	0.96 (3)	C8—H8	0.86 (2)
N2—H2A	0.82 (4)	C10—H10	0.96 (4)
N2—H2B	0.80 (3)		
			
O1—P1—O2	118.53 (10)	C6—N4—H4A	123.5 (19)
O1—P1—O3	106.68 (11)	C6—N4—H4B	117 (2)
O1—P1—O4	102.68 (10)	N1—C1—N2	119.7 (2)
O2—P1—O3	112.61 (10)	N2—C1—C2	123.0 (2)
O2—P1—O4	114.52 (10)	N1—C1—C2	117.3 (2)
O3—P1—O4	99.74 (9)	C1—C2—C3	120.3 (2)
O4—P2—O5	108.10 (10)	C2—C3—C4	119.9 (3)
O4—P2—O6	110.40 (9)	C3—C4—C5	119.5 (2)
O4—P2—O7^i^	98.08 (9)	Cl1—C4—C3	120.7 (2)
O5—P2—O6	119.81 (10)	Cl1—C4—C5	119.78 (19)
O5—P2—O7^i^	108.31 (10)	N1—C5—C4	119.6 (2)
O6—P2—O7^i^	109.93 (10)	C1—C2—H2	117.0 (19)
O3—P3—O7	99.05 (10)	C3—C2—H2	123 (2)
O3—P3—O8	109.32 (10)	C2—C3—H3A	121.8 (19)
O3—P3—O9	109.78 (11)	C4—C3—H3A	118.2 (19)
O7—P3—O8	105.20 (10)	C4—C5—H5	126 (2)
O7—P3—O9	111.56 (10)	N1—C5—H5	114 (2)
O8—P3—O9	119.86 (10)	N3—C6—C7	117.59 (19)
P1—O3—P3	134.02 (13)	N3—C6—N4	120.0 (2)
P1—O4—P2	132.54 (10)	N4—C6—C7	122.37 (18)
P2^i^—O7—P3	126.34 (11)	C6—C7—C8	119.8 (2)
P1—O1—H1A	121 (3)	C7—C8—C9	120.5 (2)
C1—N1—C5	123.4 (2)	C8—C9—C10	119.4 (2)
C5—N1—H1	119.3 (19)	Cl2—C9—C8	119.51 (19)
C1—N1—H1	117.3 (19)	Cl2—C9—C10	121.1 (2)
C1—N2—H2A	121 (2)	N3—C10—C9	119.5 (2)
H2A—N2—H2B	117 (3)	C6—C7—H7	117.6 (18)
C1—N2—H2B	119 (3)	C8—C7—H7	122.6 (18)
C6—N3—C10	123.1 (2)	C7—C8—H8	124.7 (18)
C6—N3—H3	115 (3)	C9—C8—H8	114.7 (18)
C10—N3—H3	122 (3)	N3—C10—H10	116 (2)
H4A—N4—H4B	120 (3)	C9—C10—H10	125 (2)
			
O1—P1—O3—P3	89.53 (18)	C5—N1—C1—C2	0.6 (4)
O2—P1—O3—P3	−42.1 (2)	C1—N1—C5—C4	0.8 (4)
O4—P1—O3—P3	−163.97 (16)	C10—N3—C6—N4	−177.2 (2)
O1—P1—O4—P2	−174.03 (14)	C10—N3—C6—C7	2.8 (3)
O2—P1—O4—P2	−44.19 (17)	C6—N3—C10—C9	−0.8 (4)
O3—P1—O4—P2	76.27 (15)	N1—C1—C2—C3	−1.3 (4)
O5—P2—O4—P1	144.47 (14)	N2—C1—C2—C3	179.1 (3)
O6—P2—O4—P1	11.66 (17)	C1—C2—C3—C4	0.7 (4)
O7^i^—P2—O4—P1	−103.18 (15)	C2—C3—C4—Cl1	−178.5 (2)
O4—P2—O7^i^—P3^i^	159.29 (13)	C2—C3—C4—C5	0.7 (4)
O5—P2—O7^i^—P3^i^	−88.53 (15)	Cl1—C4—C5—N1	177.8 (2)
O6—P2—O7^i^—P3^i^	44.08 (16)	C3—C4—C5—N1	−1.4 (4)
O7—P3—O3—P1	134.37 (16)	N3—C6—C7—C8	−1.8 (3)
O8—P3—O3—P1	24.7 (2)	N4—C6—C7—C8	178.1 (2)
O9—P3—O3—P1	−108.72 (17)	C6—C7—C8—C9	−1.0 (4)
O3—P3—O7—P2^i^	102.22 (14)	C7—C8—C9—Cl2	−176.3 (2)
O8—P3—O7—P2^i^	−144.80 (13)	C7—C8—C9—C10	3.0 (4)
O9—P3—O7—P2^i^	−13.34 (18)	Cl2—C9—C10—N3	177.2 (2)
C5—N1—C1—N2	−179.8 (3)	C8—C9—C10—N3	−2.1 (4)

Symmetry code: (i) −*x*, −*y*+1, −*z*+1.

### Hydrogen-bond geometry (Å, º)

**Table:**

*D*—H···*A*	*D*—H	H···*A*	*D*···*A*	*D*—H···*A*
N1—H1···O5^ii^	0.96 (3)	1.78 (3)	2.736 (3)	177 (3)
O1—H1*A*···O8^iii^	0.78 (5)	1.66 (5)	2.418 (3)	165 (5)
N2—H2*A*···O6^ii^	0.82 (4)	2.02 (4)	2.844 (3)	173 (3)
N2—H2*B*···O9^i^	0.80 (3)	2.29 (3)	3.000 (3)	149 (4)
N3—H3···O2	0.78 (4)	2.03 (3)	2.781 (3)	161 (3)
N4—H4*A*···O2	0.80 (3)	2.57 (3)	3.179 (3)	134 (2)
N4—H4*A*···O6	0.80 (3)	2.16 (3)	2.827 (3)	142 (3)
N4—H4*B*···O9^iv^	0.93 (4)	1.95 (4)	2.852 (3)	162 (3)
C5—H5···O5^v^	0.87 (3)	2.51 (3)	3.322 (3)	157 (3)
C10—H10···O1^iii^	0.96 (4)	2.42 (4)	3.262 (3)	146 (3)

Symmetry codes: (i) −*x*, −*y*+1, −*z*+1; (ii) −*x*, −*y*, −*z*+1; (iii) −*x*+1, −*y*+1, −*z*+1; (iv) *x*, *y*−1, *z*; (v) *x*, *y*, *z*−1.
